# Supplementary material for: White pupae phenotype of tephritids is caused by parallel mutations of a MFS transporter
Source: Nat Commun. 2021 Jan 21;12:491. doi: 10.1038/s41467-020-20680-5 (PMC7820335; doi:10.1038/s41467-020-20680-5)
Supplement: Supplementary file 1 — Supplementary Information [file 41467_2020_20680_MOESM1_ESM.pdf]

White pupae phenotype of tephritids is caused by parallel mutations of a MFS transporter

Ward and Aumann *et al.*

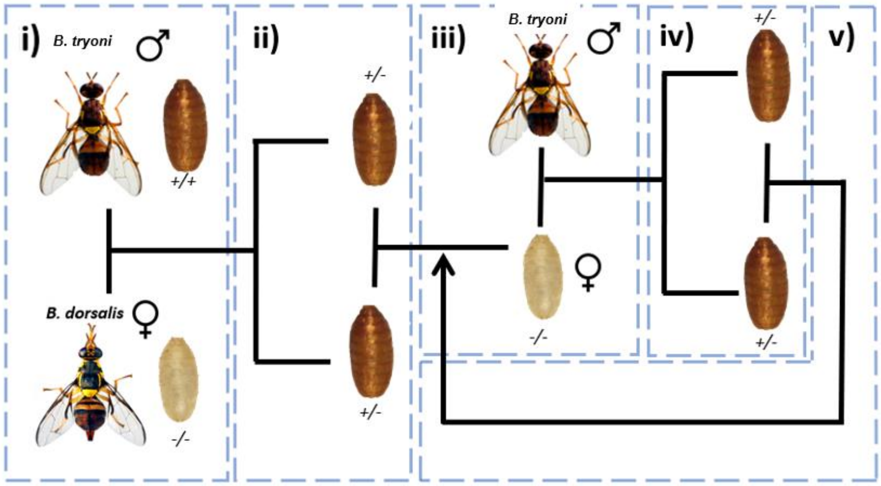

Supplementary Fig. 1. Introgression of the *B. dorsalis white pupae* locus and phenotype into *B. tryoni*. Stepwise methods are shown: i) Mass crosses between *B. tryoni* males and white pupae *B. dorsalis* females, ii) mass intercross of the F_1 progeny, iii) F_2 brown pupae were discarded and white pupae females were selectively backcrossed to *B. tryoni* males, iv) mass intercross progeny and v) reselect white pupae females and repeat backcrossing from step (iii) five additional times to produce the *Bactrocera Introgression Line* (*BIL*) with a white pupae phenotype.

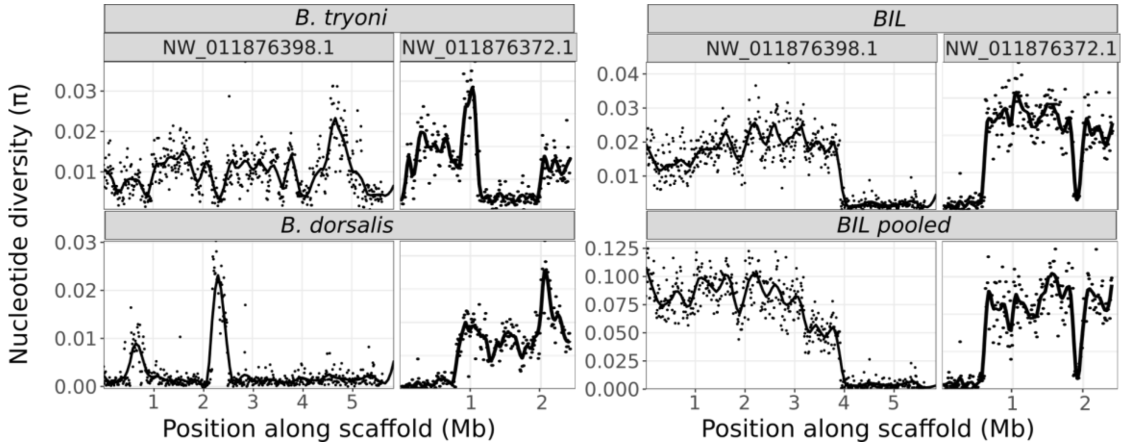

Supplementary Fig. 2. Nucleotide diversity across two genomic scaffolds belonging to chromosome 5 (NW_011876398.1; 5.8 Mb and NW_011876372.1; 2.8 Mb) containing the *Bactrocera dorsalis white pupae* QTL. Calculations were carried out on resequenced genomes mapped to the *B. dorsalis* reference genome (GCF_000789215.1) treating each strain/species as a population. Upper left: *Bactrocera tryoni* Ourimbah strain individual pupa (wild type brown pupae, n = 2). Lower left: *Bactrocera dorsalis* Salaya1 strain (white pupae, n = 2). Upper right: *Bactrocera Introgression Line* (*BIL*, white pupae, n = 2). Lower right: pooled *BIL* individuals with two replicates of five samples per pool (white pupae). White pupae populations, *B. dorsalis*, *BIL* and *BIL pooled* show low levels of diversity between 3.8 - 5.8 Mb on NW_011876398.1 and 0 - 0.73 Mb, indicating the causal mutation is within this region.

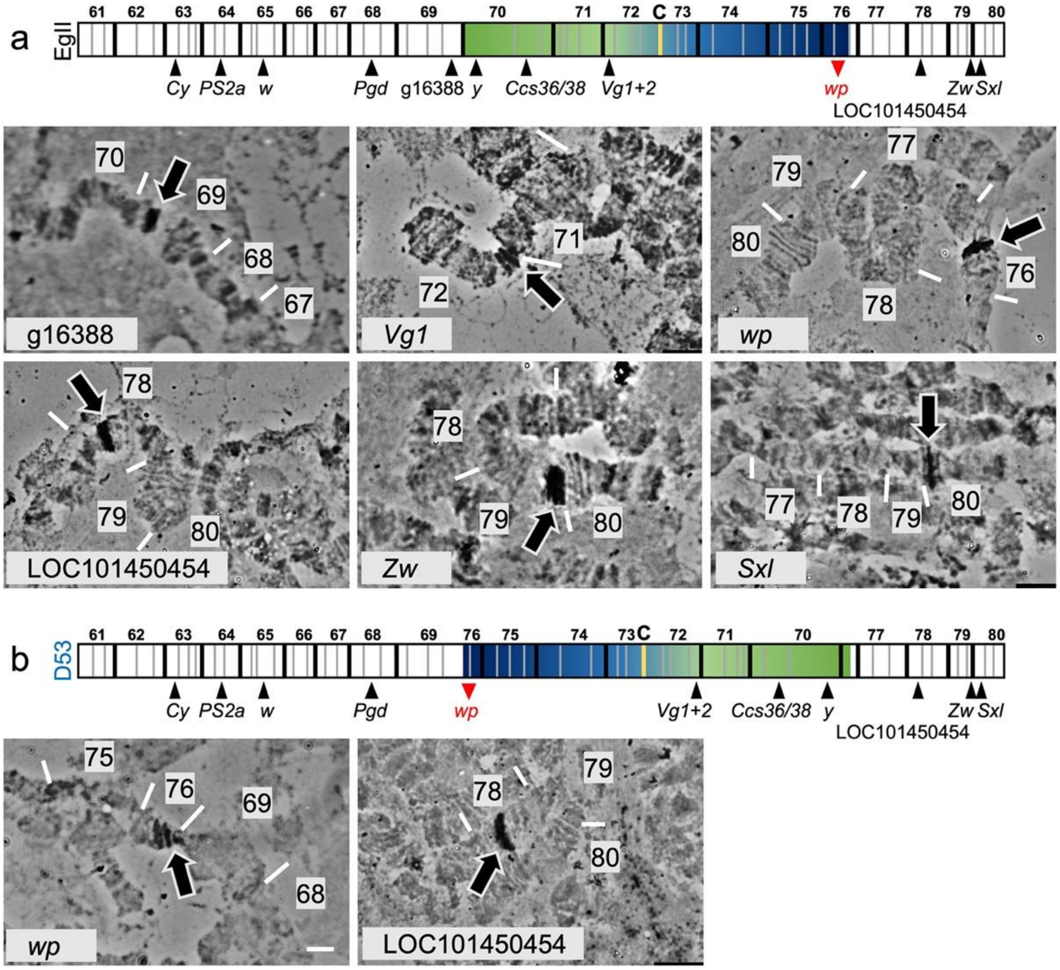

c

**Probe / gene**	**position (polytene map)**	**Start codon on scaffold_5, Ccap3.2.1**	**primers**
g16388	69D	28,345,706	P1821/1822
Vg1	72A	41,652,799	vg1_probe_F-R
wp	76B	61,551,260	P1633/1634
LOC101450454	78A	64,629,210	P1790/1791
Zw	79C	67,751,598	zw_probe_F-R
Sxl	80A	68,132,125	Sxl_probe_F-R

Supplementary Fig. 3. *In situ* hybridization of chromosome 5 marker genes in *Ceratitis capitata*. Known marker genes (*vitellogenin-1-like* (*Vg1*), *white pupae* (*wp*), *glucose-6-phosphate 1-dehydrogenase* (*Zw*), and *sex lethal* (*Sxl*)), and genes inside and outside the predicted inversion breakpoints (g16388 and LOC101450454) were used to confirm the overall structure of scaffold 5 (Ccap3.2.1; accession GCA_905071925) and the D53 inversion in WT EgII (a) and D53 (b). Chromosome 5 is shown as schematic illustration, including the additional marker genes *Curly* (*Cy*), *integrin-aPS2* (*PS2a*), *white* (*w*), *6-phosphogluconate dehydrogenase* (*Pgd*), *yellow* (*y*), and *chorion S36/38* (*Ccs36/38*). The inverted part (D53) is shown in a green-to-blue color gradient and the centromere is marked as ‘C’. Exact position of the genes on the polytene map, the position of the start codons on EgII scaffold_5 in Ccap3.2.1 and primers used to amplify the probes for *in situ* hybridization are named in (c), primer sequences are shown in Supplementary Table 5. *In situ* hybridizations were done at least in duplicates and at least ten nuclei were analyzed per sample, scale bar = 10 µm. All replicates led to similar results. The source data underlying Supplementary Figure 3a and 3b are provided as a Source Data file.

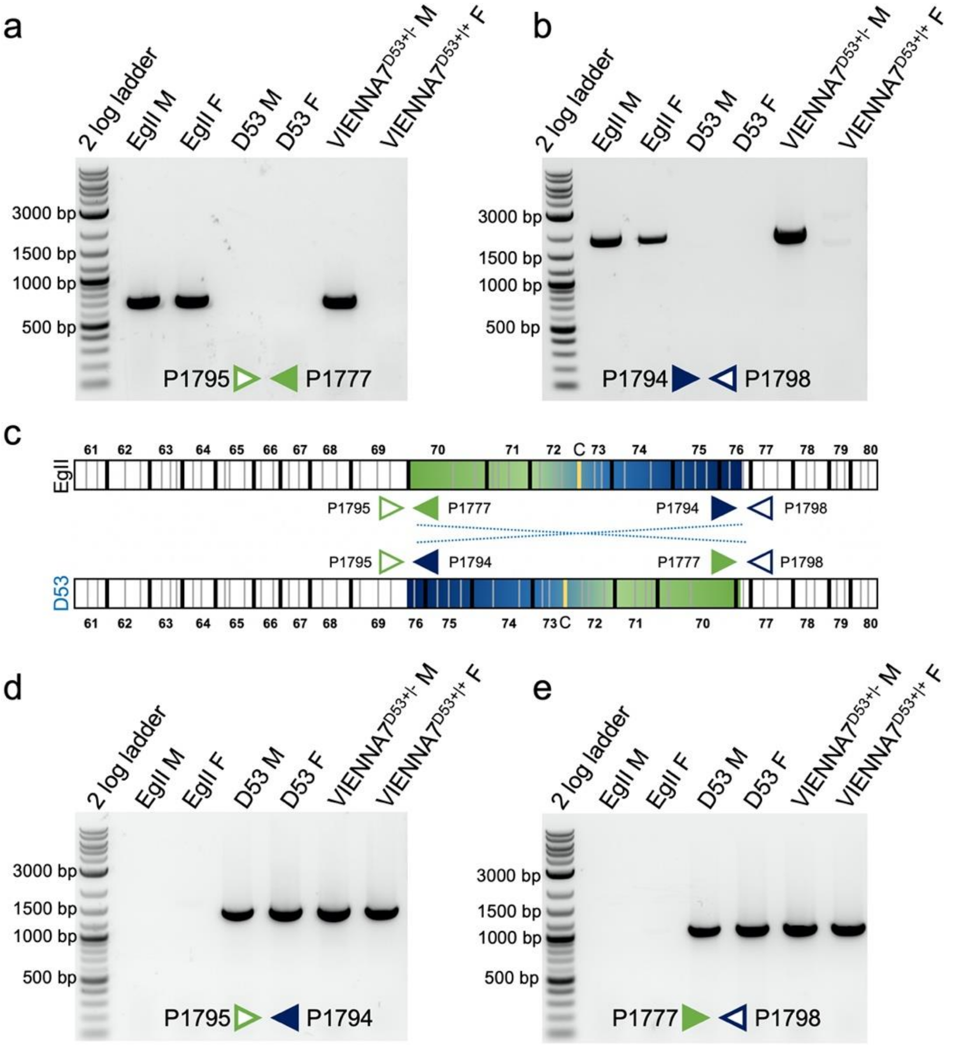

Supplementary Fig. 4. Verification of the D53 inversion breakpoints via PCRs. Two primer pairs were used to amplify the sequences spanning the inversion breakpoints in EgII (WT), D53 (homozygous for the inversion), and VIENNA7^{D53+} males (heterozygous for the inversion) and females (homozygous for the inversion). Schematic primer positions are shown in (c) (indicated by triangles), together with schematics of the chromosome 5 without (top) and with (bottom) the D53 inversion, which is shown as a reversed blue-to-green color gradient. The centromere is marked as 'C'. The left breakpoint in strains without inversion was amplified with primers P_1795 and P_1777 (a), the right breakpoint with the primer pair P_1794/P_1798 (b). For the amplification of the breakpoints in strains with inversion, the internal primers were used vice versa: (d) P_1795/P_1794, (e) P_1777/P_1798. Primers P_1794 and P_1798 were designed based on EgII chromosome 5, flanking the predicted breakpoint position (61,880,224 bp; Ccap3.2.1, accession GCA_905071925). Primers P_1795 and P_1777 were designed based on D53 nanopore data (D53 assembly contig_531), as the left breakpoint is within a scaffold gap on EgII chromosome 5. The ladder used for agarose gels is the NEB 2-log DNA-ladder, bp = base pairs; M = male; F = female. PCRs were done at least twice to confirm the result; primer sequences are shown in Supplementary Table 5. The source data underlying Supplementary Figure 4a, 4b, 4d, and 4e are provided as a Source Data file.

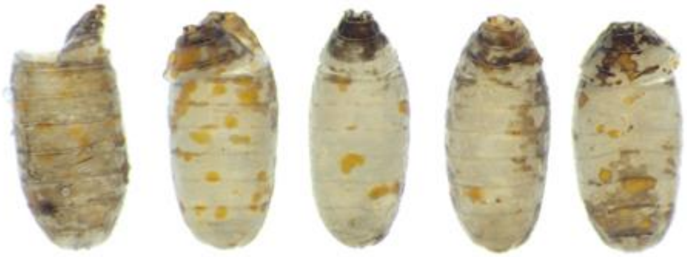

Supplementary Fig. 5. *Bactrocera tryoni* pupae exhibiting somatic mosaicism after embryonic injection of CRISPR/Cas9 guide RNA targeting exon 1 of the *white pupae* gene.

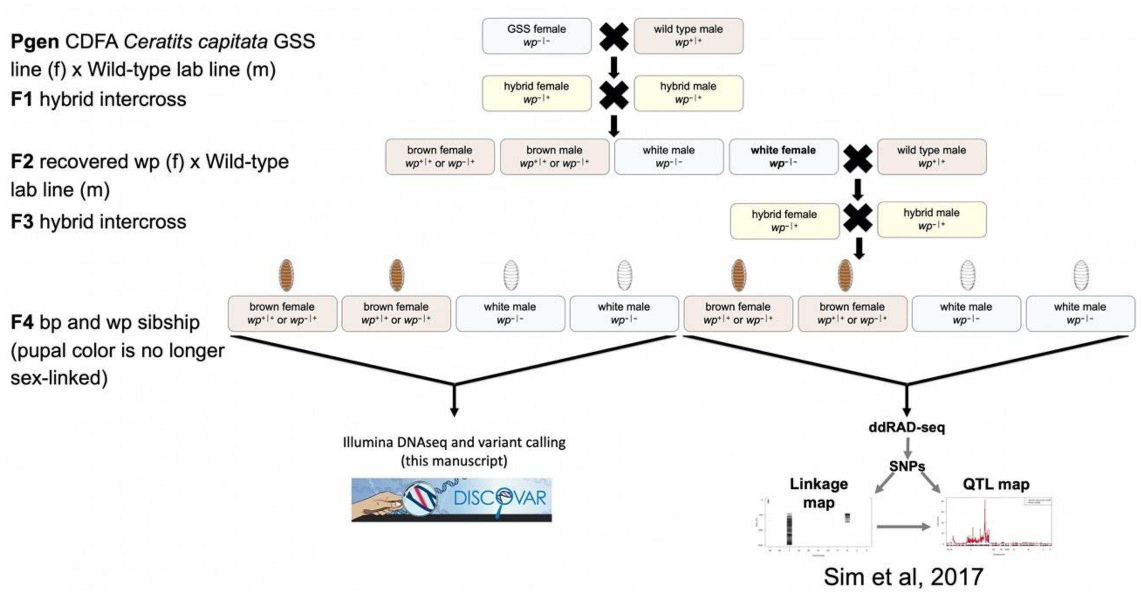

Supplementary Fig. 6. Relationship of the whole genome sequenced *C. capitata* individuals to the mapping population described in Sim *et al*[1]: utilizing pangenomic assemblies to characterize region and mutations associated with white pupae in *Ceratitis capitata*. The putative region of the medfly genome thought to contain the *white pupae* mutation was previously identified as being on NW_004523946.1 of the AOHK00000000.1 WGS accession for *C. capitata*, determined by genotyping a mapping population of a wild type strain with the Vienna genetic sexing strain[1]. However, genotyping was performed using restriction site associated genotype by sequencing, and the resolution of the map only gave resolution within several million base pairs of the actual causative mutation. To better explore putative mutations associated with the white pupae phenotype, single individual *de novo* assemblies were generated from stable white and stable brown individuals from the mapping population. Three white males ($wp^{-|-}$) (from population W8_3_2_2) and three brown females ($wp^{+|+}$ or $wp^{-|+}$) (from population B8_3_2_1) from a common Pgen and parents were sequenced using PCR free library preparation, and each was sequenced to ~50x coverage using Illumina 2 x 250 paired-end sequencing. These libraries are made public on NCBI under SRA accessions SRR11649127-SRR11649132 associated with BioProject PRJNA629430. The libraries were sized selected to allow overlap of the read pairs through double sided Ampure size selection. The subsequent data was assembled in tandem using the DISCOVAR *de-novo* assembler, using the AOHK00000000.1 WGS assembly available on NCBI to anchor all of the individual-scale assemblies to each other and call variants relative to each other[2]. This approach creates a single assembly graph, with all the individual assemblies being represented within this graph which was visualized using the NhoodInfo tool. A secondary method, using GATK 3.3.0 and the Unified Genotyper pipeline[3] was used to create a .vcf containing variants between the genomes. To target for mutations associated with the white pupae phenotype, this was screened across the scaffolds within 20 Mb of the QTL loci and filtered for assigned genotype probability >0.995. Final variants from this pipeline were filtered using vcftools[4] to subset to those that were consistent between phenotypes, identifying loci that are homozygous for one allele in white pupae individuals and homozygous for another allele or heterozygous in brown pupae individuals. SNPs between the two variant calling approaches, in graph space, and mapping base VCF were merged, and the SNPEff tool[5] was used to filter for SNPs causing high impact in protein coding regions based off of the annotation set accompanying the genome in NCBI. This resulted in a subset of variants on NW_004523946.1 that were putatively identified as causative, or directly linked to the causative mutation (Supplementary Table 3).

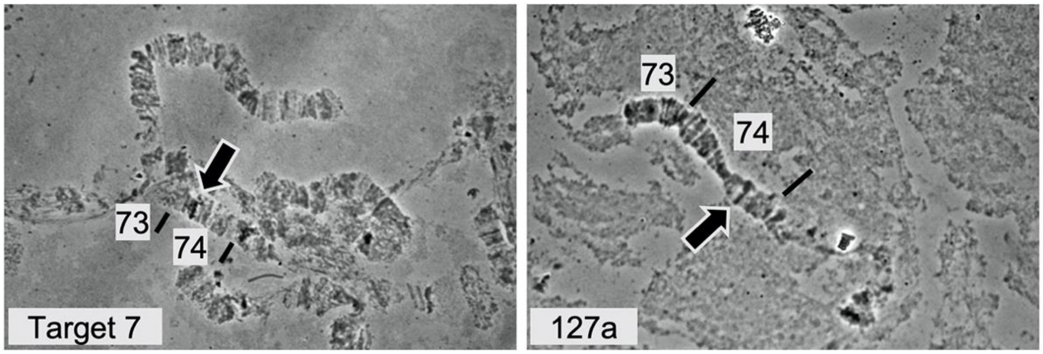

Supplementary Fig. 7. *In situ* hybridization on target 7 (see Supplementary Table 3), the region surrounding the locus with the highest LOD score and perfect LD with the white pupae phenotype, described in Sim *et al.*[1] (59,869,348 - 59,871,012 on scaffold_5, Ccap3.2.1), and 127a, a randomly chosen gene on the same scaffold in Ccap2.1 (LOC101458400; 59,396,172 - 59,397,378 on scaffold_5, Ccap3.2.1). *In situ* hybridizations were done at least in duplicates and at least ten nuclei were analyzed per sample. Pictures were taken without a scale bar and cannot be scaled. All replicates led to similar results. Source data are provided as a Source Data file.

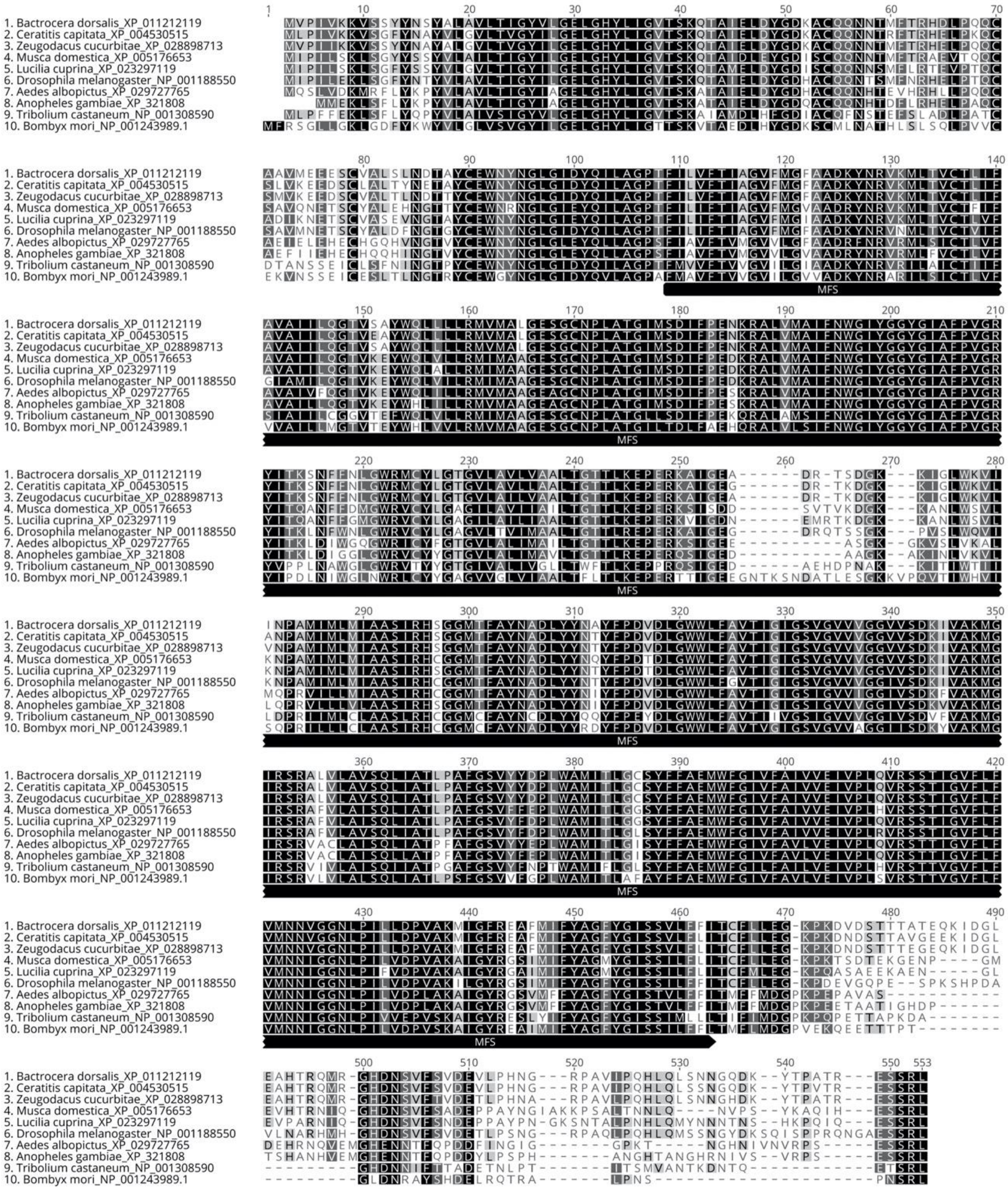

Supplementary Fig. 8. Amino acid sequence alignment of insect *white pupae* homologs using MUSCLE. Identical residues are shaded in black, conserved residues in shades of grey and the MFS domain is underlined.

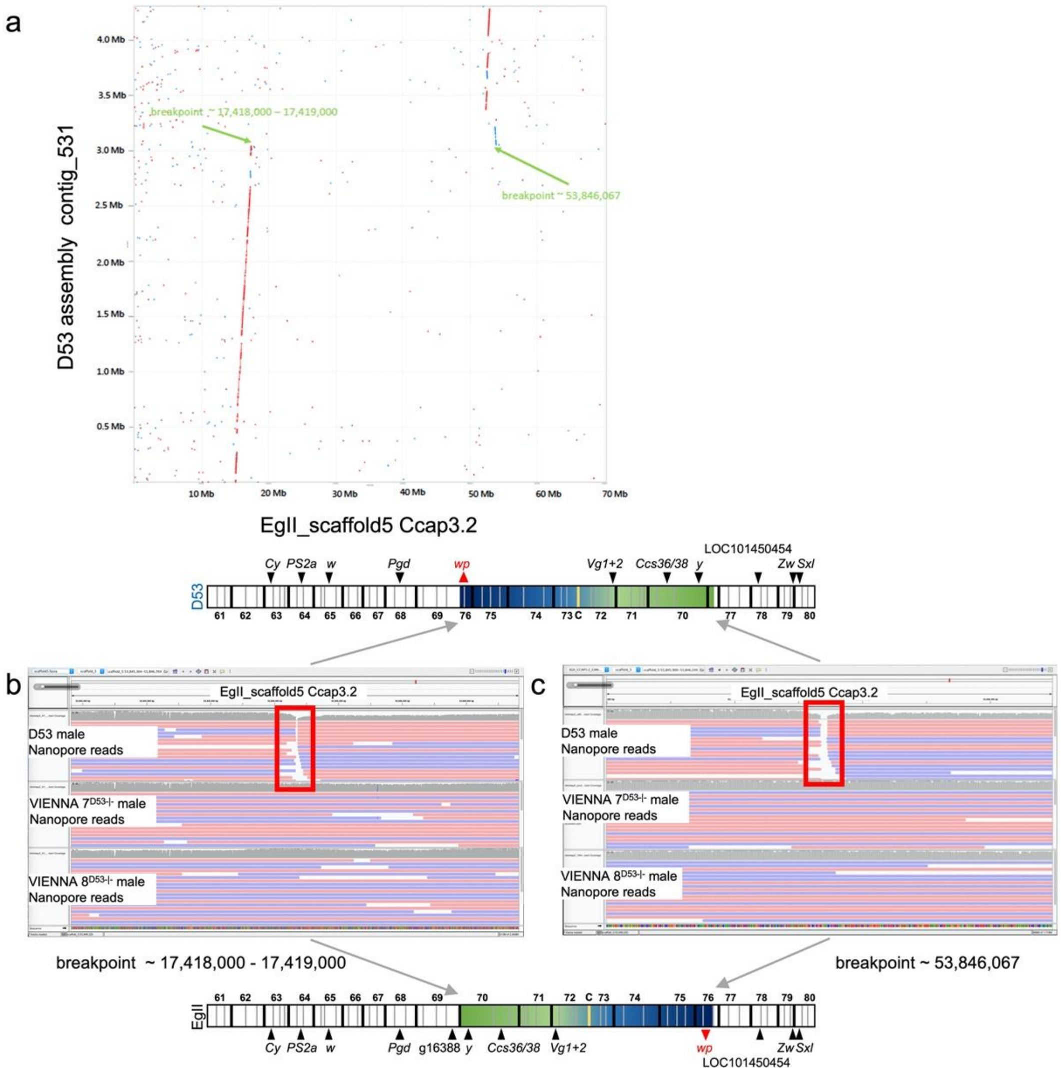

Supplementary Fig. 9. Identification of the D53 inversion breakpoints. (a) The EgII (wild type; EgII) assembly was plotted vs the D53 Nanopore assembly. The EgII Ccap 3.2 assembly scaffold_5 is shown on the x-axis compared to the D53 assembly contig 531 using the MashMap long read mapping tool, providing a visual overview of the local alignment boundaries. The D53 assembly breaks its homology with the EgII assembly at the 17.4 Mbp position of scaffold 5 and continues further at position 53.84 Mbp, providing identification of candidate breakpoints. (b)-(c): Zoomed in view using Integrative Genome Viewer (IGV, http:// software.broadinstitute.org/software/igv/) of D53 male (top) VIENNA $7^{D53-|-}$ male (middle) and VIENNA $8^{D53-|-}$ male (bottom) Nanopore reads aligned against the EgII Ccap 3.2 genome reference scaffold 5. Reads aligned in the sense direction are shown in red, reads aligning in antisense in blue. The alignments of D53 Nanopore reads (top) break in the positions of 17,418,000 for the left breakpoint on chromosome 5 (b) and at position 53,846,067 corresponding to the right breakpoint (c). However, VIENNA $7^{D53-|-}$ male and VIENNA $8^{D53-|-}$ male Nanopore reads align perfectly to the EgII reference without breaks. Annotated ideograms of the D53 inversion chromosome and the EgII wild type chromosome are shown at the top and bottom, respectively. Marker genes are described in Supplementary Fig. 3.

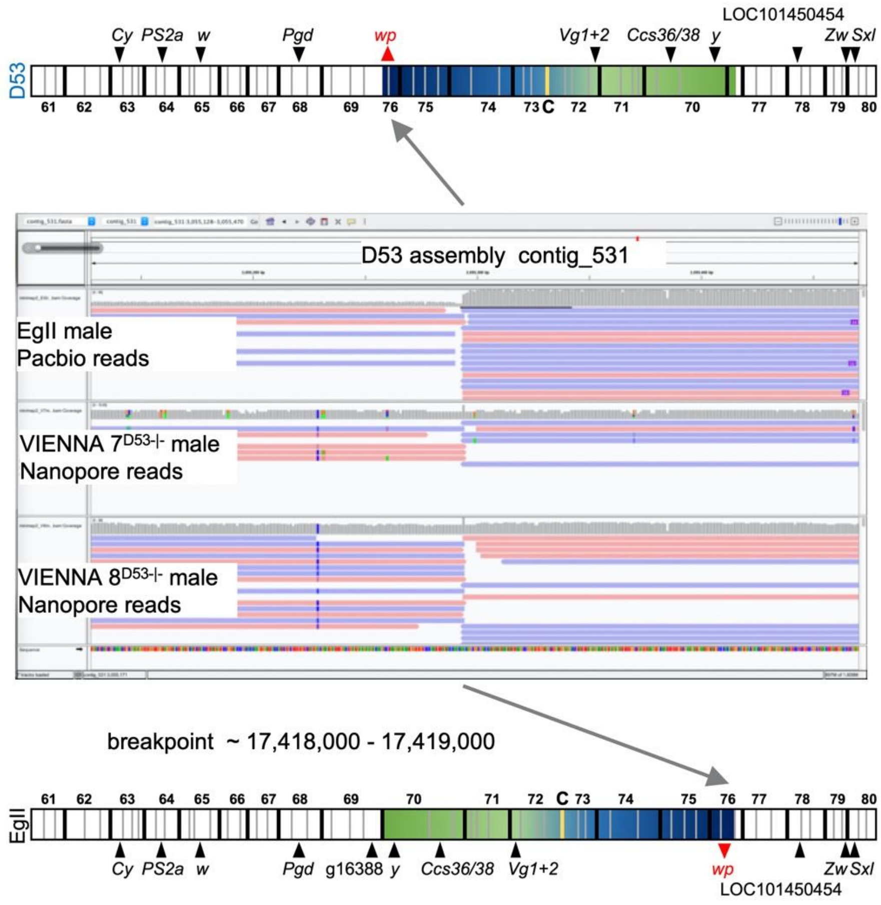

Supplementary Fig. 10. Example of D53 inversion breakpoint confirmation. Zoomed in view, using IGV, of the EgII male PacBio reads (top) VIENNA $7^{D53\text{-}|\text{-}}$ male (middle) and VIENNA $8^{D53\text{-}|\text{-}}$ male (bottom) Nanopore reads aligned against the D53 genome assembly (Nanopore reads) contig 531. Reads aligned in the sense direction are shown in red, reads aligning in antisense in blue. The alignments of EgII PacBio reads (top) break in the position of 17,418,000 for the left breakpoint on chromosome 5, along with VIENNA 7 $^{D53\text{-}|\text{-}}$ male and VIENNA 8 $^{D53\text{-}|\text{-}}$ (middle and bottom) Nanopore reads consistent with the presence of inversion only in the D53 genome. Annotated ideograms of the D53 inversion chromosome and the EgII wild type chromosome are shown at the top and bottom, respectively. Marker genes are described in Supplementary Fig. 3.

Supplementary Table 1. Strains used for genomic and transcriptomic analysis.

No.	Species	Strain	Sex	Genotype	Inver sion	Trans- location	Pupa color	**Illumina MiSeq** Chromosome dissection	**PacBio** WGS	**Hi-C** WGS	**Nanopore** WGS	**Illumina** WGS	**Illumina NovaSeq 6000** RNAseq
1	*C. capitata*	EgyptII (EgII)	Male	WT	NO	NO	brown	Y (ERS4426864), X (ERS4426865)	Yes (ERS4426857- ERS4426863)	Yes (ERS45195 15)	Yes (ERS45475 91)	Yes (ERS4426868) HiSeq 4000	
2	*C. capitata*	EgII	Female	WT	NO	NO	brown					Yes (ERS4426869) HiSeq 4000	
3	*C. capitata*	Benakeion	Larvae / prepupae	WT	NO	NO	brown						Yes (ERS4426994 - ERS4426999)
4	*C. capitata*	*wp/tsl*<br>(in EgII)	Larvae / prepupae	*wp/tsl* homo	NO	NO	white						Yes (ERS4427000 - ERS4427005)
5	*C. capitata*	D53<br>(in wp/tsl EgII)	Male	*wp/tsl* homo	YES	NO	white				Yes (ERS45475 90)	Yes (ERS4426872) HiSeq 4000	
6	*C. capitata*	D53<br>(in wp/tsl EgII)	Female	*wp/tsl* homo	YES	NO	white					Yes (ERS4426873) HiSeq 4000	
7	*C. capitata*	Vienna 7 $^{D53-}$	Male	*wp/tsl* hetero	NO	YES (Vienna 7)	brown	T(Y;5) (ERS4426866), X (ERS4426867)			Yes (ERS45475 92)	Yes (ERS4426870) HiSeq 4000	
8	*C. capitata*	Vienna 7 $^{D53-}$	Female	*wp/tsl* homo	NO	NO	white					Yes (ERS4426871) HiSeq 4000	
9	*C. capitata*	Vienna 8 $^{D53-}$	Male	*wp/tsl* hetero	NO	YES (Vienna 8)	brown				Yes (ERS45475 93)		
10	*B. dorsalis*	Saramburi	Larvae / prepupae	WT	NO	NO	brown						Yes (ERS4427006 - ERS4427011)
11	*B. dorsalis*	white pupae	Larvae / prepupae	*wp* homo	NO	NO	white						Yes (ERS4427012 - ERS4427017)

12	*B. dorsalis*	white pupae	Male	*wp* homo	NO	NO	white					Yes (SRR13206143) NovaSeq 6000	
13	*B. dorsalis*	white pupae	Female	*wp* homo	NO	NO	white					Yes (SRR13206144) NovaSeq 6000	
14	*B. tryoni*	Ourimbah	Male	WT	NO	NO	brown					Yes (SRR13206145) NovaSeq 6000	
15	*B. tryoni*	Ourimbah	Female	WT	NO	NO	brown					Yes (SRR13206146) NovaSeq 6000	
16	Hybrid	Bactrocera Introgressed Line	Male	*wp* homo	NO	NO	white					Yes (SRR13206141) NovaSeq 6000	
17	Hybrid	Bactrocera Introgressed Line	Male	*wp* homo	NO	NO	white					Yes (SRR13206142) NovaSeq 6000	
18	Hybrid	Bactrocera Introgressed Line	Pooled males	*wp* homo	NO	NO	white					Yes (SRR13206139) NovaSeq 6000	
19	Hybrid	Bactrocera Introgressed Line	Pooled Females	*wp* homo	NO	NO	white					Yes (SRR13206140) NovaSeq 6000	
20	*Z. cucurbitae*	wp GSS	Larvae / prepupae	Mixed. Individual preps. Sex, and by extrapolation the pupal color, was confirmed by *MoY* PCR	NO	YES	brown/ white						Yes (brown: ERS4427018 - ERS4427023; white: ERS4427024 - ERS4427029)

Note: Accession numbers ENA BioProject PRJEB36344/ERP119522, and BioProject PRJNA682907.

Supplementary Table 2. Illumina pair-end sequencing of *Ceratitis capitata* strains and micro-dissected chromosomes.

**Strain**	**Chromosome (N) / Sex**	**Coverage**	**Accession number**
Egypt II	Y (20)	~7 x	ERS4426864
Egypt II	X (20)	~6 x	ERS4426865
VIENNA 7^{D53-}	T(Y;5) (25)	~6 x	ERS4426866
VIENNA 7^{D53-}	X (20)	~8 x	ERS4426867
Egypt II	Male	~40 x	ERS4426868
Egypt II	Female	~45 x	ERS4426869
VIENNA 7^{D53-}	Male	~35 x	ERS4426870
VIENNA 7^{D53-}	Female	~30 x	ERS4426871
D53	Male	~32 x	ERS4426872
D53	Female	~27 x	ERS4426873

Note: Third instar larvae mitotic chromosomes were laser micro-dissected[6,7] and used to construct libraries using the PicoPLEX WGA kit. These libraries were then multiplexed into a single run of Illumina Miseq platform for 250 bp paired-end sequencing. For the whole genome sequencing of Egypt II, VIENNA 7^{D53-} and D53 strains, fresh pupae (1 day after pupation) were used as the insect material for DNA extraction using the phenol/chloroform approach (see methods). This DNA was analyzed for size and quality. Single insects (males or females) were used from each of the three insect lines to make 6 indexed Illumina TruSeq PCR-free libraries (550 bp inserts) from the extracted genomic DNA. These libraries were then multiplexed into one lane of the Illumina HiSeq 4000 150 bp platform for paired-end sequencing. Sequence data are available at: ENA BioProject accession number PRJEB36344/ERP119522.

Supplementary Table 3. Position of high impact variants identified using the union of the DISCOVAR and GATK genotyping methods performed on WGS data from phenotypic white and brown F4 *Ceratitis capitata* individuals from the mapping population described in Sim *et al*[1] (see Supplementary Fig. 6). No. 7 is described as the SNP locus displaying the strongest linkage to the wp phenotype.

No.	Scaffold	Position	Ref	Alt
1	NW_004523946.1	800831	C	T
2	NW_004523946.1	837972	C	C
3	NW_004523946.1	1576424	G	A
4	NW_004523946.1	2259830	AAC	A
5	NW_004523946.1	2262779	C	A
6	NW_004523946.1	2410888	A	ACAACAGGCATGCCAGCAAGTTGT GGCCGTCTTCCAACAACATGCTGCT ACAACTACAACAGCCAAATGACGA GCCCGCCGTTGCAGCCTCAGCACCA GCCAAGGCTACATATGCAACACTGC GACATGCGATGGTTGTAGAGGCGC AAGC
7	NW_004523946.1	1353742	G	T

Supplementary Table 4. Nanopore sequencing run metrics.

**Sample**	**Flow cell ID**	**Pores**	**Library kit**	**Bases (Gb)**	**N50 (kb)**	**MinKNOW PromethION Release**	**Guppy**
Egypt II male	PAD68071	2827	SQK-LSK109	7.64	6.82	19.06.9	3.0.3
Egypt II male	PAD68071	159	SQK-RAD004	0.06	0.87	19.06.9	3.0.3
D53 male	PAD66870	6288	SQK-LSK109	19.88	14.04	19.06.9	3.0.3
D53 male	PAD66870	2991	SQK-RAD004	6.42	6.73	19.06.9	3.0.3
D53 male	PAD75166	2081	SQK-LSK109	4.79	0.91	19.10.2	3.2.6
VIENNA 7^{D53-} male	PAD60029	25	SQK-LSK109	0.21	4.86	19.06.9	3.0.3
VIENNA 7^{D53-} male	PAD73592	3270	SQK-LSK109	7.47	5.37	19.10.2	3.2.6
VIENNA 7^{D53-} male	PAE49665	5025	SQK-LSK109	9.34	5.59	19.12.5	3.2.8
VIENNA 7^{D53-} male	PAE47816	3749	SQK-LSK109	5.73	6.42	19.12.5	3.2.8
VIENNA 7^{D53-} male	PAE47455	1503	SQK-RAD004	3.97	3.88	19.12.5	3.2.8
VIENNA 8^{D53-} male	PAE03818	3557	SQK-LSK109	7.68	6.22	19.06.9	3.0.3
VIENNA 8^{D53-} male	PAD88883	3209	SQK-LSK109	7.02	6.19	19.10.2	3.2.6
VIENNA 8^{D53-} male	PAE16370	2473	SQK-LSK109	6.90	5.73	19.10.2	3.2.6
VIENNA 8^{D53-} male	PAE47104	3143	SQK-RAD004	3.23	3.41	19.12.5	3.2.8

Supplementary Table 5. List of primers used in this study.

Name	Sequence	Purpose
P_1753	GAAATTAATACGACTCACTATAGGCAT GCCGCCAGAGTGACGAAGTTTTAGAG CTAGAAATAGC	gRNA_MFS (*C. capitata*) *in vitro* synthetization
P_369	GCACCGACTCGGTGCCACTTTTTCAA GTTGATAACGGACTAGCCTTATTTTAA CTTGCTATTTCTAGCTCTAAAAC	gRNA_MFS (*C. capitata*) *in vitro* synthetization
P_1643	TTGAAGAGCGCACTTGCAAC	*Cc_wp* non-lethal genotyping G_1
P_1644	TTCCCCAACAGTGAATCCGG	*Cc_wp* non-lethal genotyping G_1
P_1657	AAACGCTCTACAGATTGTGGA	multiplex PCR non-lethal genotyping G_2
P_1794	ATCTACCAAATGAGAGAGAGAGCG	D53 inversion verification
P_1795	TTTTTGAAACCACTTGAACAACGC	D53 inversion verification
P_1777	TCCAGTGTTCTCTACTATGTTGCC	D53 inversion verification
P_1798	TCAGCTAACAGAACATGAATTCCG	D53 inversion verification
BtMFS_5primeF	TTTTTGCTTATCCCACTTCTGATT	PCR amplicons spanning both *BtMFS* guide recognition sites
BtMFS_exon2R	ACACCAGCAATTGTAAAGACCA	PCR amplicons spanning both *BtMFS* guide recognition sites
ZcMoY1F	AAGCCAGATCACGCAATCC	maleness-specific PCR on the *MoY* gene of *Z. cucurbitae*
ZcMoY1R	AGGACATCGTTATCTCCCCTG	maleness-specific PCR on the *MoY* gene of *Z. cucurbitae*
Bt_MFS-1	TGTGAGTACGGCCAACGCAT	customized 20 bp crRNA sequence
Bt_MFS-2	CGATCTACCACAGCAATGTG	customized 20 bp crRNA sequence
P_1790	AATCAAGTAAAGACAAAGCGGACG	*in situ* probe ‚*Cc*_LOC101450454 ‘
P_1791	TCATACGAACAGTTTGCCATAACG	*in situ* probe ‚*Cc*_LOC101450454 ‘
P_1821	TAAATGATTTGTCCGCTGAAGCC	*in situ* probe ‚*Cc*_g16388‘
P_1822	GAACACTATCCATGCTCTTGTCC	*in situ* probe ‚*Cc*_g16388‘
P_1395	TCTCTGGGCAGCTCAAAGTG	*in situ* probe ‚*Cc*_target 7‘
P_1396	AAACCAAACATTGCGGGCTC	*in situ* probe ‚*Cc*_target 7‘
P_1415	TCTCCCACACACTCAGGTCA	*in situ* probe ‚*Cc*_127a‘
P_1416	ACTCTCGTTGTCTGCTTGCA	*in situ* probe ‚*Cc*_127a‘
P_1633	TCCAGTGCAGTTCGGCTTAA	*in situ* probe *Cc_wp*
P_1634	CGGCTTTTACAACGCTTATGTTC	*in situ* probe *Cc_wp*
Zc_F	GTCATGACCACGCATTTGACG	*in situ* probe *Zc_wp*
Zc_R	GTTTATGGGTTTCGCCGCTG	*in situ* probe *Zc_wp*

Name	Sequence	Purpose
Bd_F	GCTGTTGCTGTTGCGTATGG	*in situ* probe *Bd_wp*
Bd_R	GTGGCGGGCGTATATTTGTC	*in situ* probe *Bd_wp*
Pgd_probe_F	TTGCTTTCTCTCCTTCTGCTT	*in situ* probe *Cc_Pgd*
Pgd_probe_R	TTCAAGTACTCACAAACGCTTGA	*in situ* probe *Cc_Pgd*
vg1_probe_F	ATCTCCGATAATCTCACAGGAAAT	*in situ* probe *Cc_Vg1*
vg1_probe_R	TCAGAGCGGGTCCATCGAAT	*in situ* probe *Cc_Vg1*
Sxl_probe_F	GCCAAAGAGATTGTTGTGTCAC	*in situ* probe *Cc_Sxl*
Sxl_probe_R	AAATTCCTGCACTGCGCTGT	*in situ* probe *Cc_Sxl*
y_probe_F	CCGTCGCCACTGTTGCTATT	*in situ* probe *Cc_y* (LOC101455502)
y_probe_R	AGTCGGGGTTGGTTGTTGTT	*in situ* probe *Cc_y* (LOC101455502)
zw_probe_F	TGCACACTGCTGCCATAGAT	*in situ* probe *Cc_Zw*
zw_probe_R	AGCCTCCTTAGCGGTTACAC	*in situ* probe *Cc_Zw*

Supplementary References

1 Sim, S. B., Ruiz-Arce, R., Barr, N. B. & Geib, S. M. A new diagnostic resource for *Ceratitis capitata* strain identification based on QTL mapping. *G3 (Bethesda)* **7,** 3637-3647 (2017).

2 Love, R. R., Weisenfeld, N. I., Jaffe, D. B., Besansky, N. J. & Neafsey, D. E. Evaluation of DISCOVAR *de novo* using a mosquito sample for cost-effective short-read genome assembly. *BMC Genomics* **17,** 187 (2016).

3 Poplin, R. *et al.* Scaling accurate genetic variant discovery to tens of thousands of samples. *bioRxiv***,** 201178 (2018).

4 Danecek, P. *et al.* The variant call format and VCFtools. *Bioinformatics* **27,** 2156-2158 (2011).

5 Cingolani, P. *et al.* A program for annotating and predicting the effects of single nucleotide polymorphisms, SnpEff: SNPs in the genome of *Drosophila melanogaster* strain w1118; iso-2; iso-3. *Fly (Austin)* **6,** 80-92 (2012).

6 Fukova, I. *et al.* Probing the W chromosome of the codling moth, *Cydia pomonella*, with sequences from microdissected sex chromatin. *Chromosoma* **116,** 135-145 (2007).

7 Kubickova, S., Cernohorska, H., Musilova, P. & Rubes, J. The use of laser microdissection for the preparation of chromosome-specific painting probes in farm animals. *Chromosome Res* **10,** 571-577 (2002).
