# Supplementary material for: White pupae phenotype of tephritids is caused by parallel mutations of a MFS transporter
Source: Nat Commun. 2021 Jan 21;12:491. doi: 10.1038/s41467-020-20680-5 (PMC7820335; doi:10.1038/s41467-020-20680-5)
Supplement: Supplementary file 2 — Reporting Summary [file 41467_2020_20680_MOESM2_ESM.pdf]

nature research

Corresponding author(s): M. F. Schetelig

Last updated by author(s): Dec 6, 2020

Reporting Summary

Nature Research wishes to improve the reproducibility of the work that we publish. This form provides structure for consistency and transparency in reporting. For further information on Nature Research policies, see our Editorial Policies and the Editorial Policy Checklist.

Statistics

For all statistical analyses, confirm that the following items are present in the figure legend, table legend, main text, or Methods section.

n/a	Confirmed	
☐	☒	The exact sample size (*n*) for each experimental group/condition, given as a discrete number and unit of measurement
☒	☐	A statement on whether measurements were taken from distinct samples or whether the same sample was measured repeatedly
☒	☐	The statistical test(s) used AND whether they are one- or two-sided<br>*Only common tests should be described solely by name; describe more complex techniques in the Methods section.*
☒	☐	A description of all covariates tested
☒	☐	A description of any assumptions or corrections, such as tests of normality and adjustment for multiple comparisons
☒	☐	A full description of the statistical parameters including central tendency (e.g. means) or other basic estimates (e.g. regression coefficient) AND variation (e.g. standard deviation) or associated estimates of uncertainty (e.g. confidence intervals)
☒	☐	For null hypothesis testing, the test statistic (e.g. F, t, r) with confidence intervals, effect sizes, degrees of freedom and P value noted<br>*Give P values as exact values whenever suitable.*
☒	☐	For Bayesian analysis, information on the choice of priors and Markov chain Monte Carlo settings
☒	☐	For hierarchical and complex designs, identification of the appropriate level for tests and full reporting of outcomes
☒	☐	Estimates of effect sizes (e.g. Cohen's *d*, Pearson's *r*), indicating how they were calculated

Our web collection on statistics for biologists contains articles on many of the points above.

Software and code

Policy information about availability of computer code

Data collection

No custom code was used for data collection.

(Sequence information was obtained from NCBI (https://www.ncbi.org/) or gained from own sequencing approaches. Sanger sequencing to analyse amplicons of C. capitata white pupae mutants and to analyze the D53 inversion breakpoints was performed by Macrogen Europe. Images of B. tryoni pupae were taken with an Olympus SZXI6 microscope, Olympus DP74 camera and Olympus LF-PS2 light source using the Olympus stream basic 2.3.3 software. Images of C. capitata pupae were taken with a Keyence digital microscope VHX-5000. For gel documentation, the automated imaging system VersaDoc MP Molecular Imager (BioRad) was used. For analysis of in situ hybridization sites, a Leica DM 2000 LED microscope and a Leica DMC 5400 camera was operated with the LAS X software 3.7.0.)

Software used in this study:
Olympus stream basic 2.3.3
LAS X v3.7.0
FastQC v0.11.6
ngsReports v1.3
Trimmomatic v0.38
NextGenMap v0.5.5
Picard v2.2.4
Freebayes v1.0.2
BCFtools v1.9
SeqArray v1.26.2
geaR v0.1
BUSCO v3

RAxML v8.2.10
Astral III v5.1.1
Salsa v2.2
3D de novo assembly (3D-DNA) pipeline version 180419
Trinity Release v2.8.5, May 2019
STAR STAR_2.5.2a
CANU v1.8
haploMerger2 v.20161205
Funannotate v1.6.0-24f34f6
SAMtools v1.9
MaSuRCA v3.3
Geneious v11
Geneious Prime
MashMap v2.0
minimap2 v2.17

Data analysis

During the analysis we used published or open source software as well as commercial software, all tools have been referenced in text.

For manuscripts utilizing custom algorithms or software that are central to the research but not yet described in published literature, software must be made available to editors and reviewers. We strongly encourage code deposition in a community repository (e.g. GitHub). See the Nature Research guidelines for submitting code & software for further information.

Data

Policy information about availability of data

All manuscripts must include a data availability statement. This statement should provide the following information, where applicable:

- Accession codes, unique identifiers, or web links for publicly available datasets
- A list of figures that have associated raw data
- A description of any restrictions on data availability

Data supporting the findings of this work are available within the paper and its Supplementary Information files. All sequence libraries prepared during this study are publicly available on NCBI within the ENA BioProject PRJEB36344//ERP119522 [https://www.ncbi.nlm.nih.gov/bioproject/PRJEB36344; https://trace.ncbi.nlm.nih.gov/Traces/sra/?study=ERP119522] (accession numbers ERS4426857 - ERS4426873, ERS4426994 - ERS4427029, ERS4519515, ERS4547590 - ERS4547593; see Supplementary Table 1), The BioProject PRJNA682907 (https://www.ncbi.nlm.nih.gov/bioproject/682907) (SRR13206139 - SRR13206144, SRR13206146 - SRR13206147; BIL WGS; see Supplementary Table 1), and the BioProject PRJNA629430 [https://www.ncbi.nlm.nih.gov/bioproject/PRJNA629430] (SRA accessions SRR11649127 - SRR11649132; see Supplementary Fig. 7). The genome assembly Ccap3.2.1 is available on NCBI, accession GCA_905071925 [https://www.ncbi.nlm.nih.gov/assembly/GCA_905071925.1/].
The datasets and insect strains generated and analyzed during the current study are available from the corresponding authors upon request. The source data underlying Figures 1 a-g, 2c-f, 3d-f, 4d, and Supplementary Figures 3a-b, 4a-b, 4d-e, and 8, are provided as a Source Data file.

Field-specific reporting

Please select the one below that is the best fit for your research. If you are not sure, read the appropriate sections before making your selection.

- [x] Life sciences
- [ ] Behavioural & social sciences
- [ ] Ecological, evolutionary & environmental sciences

For a reference copy of the document with all sections, see nature.com/documents/nr-reporting-summary-flat.pdf

Life sciences study design

All studies must disclose on these points even when the disclosure is negative.

Sample size

Sample size numbers are referenced in text.

Sequencing
- WGS is based on selected samples and sample size calcuations are not relevant for signifcance. Single flies would indeed lead to the most significant results if technology and DNA isolations allow for such approaches. Therefore, sample size calcualtions for WGS are not relevant.
The sample sizes used for the experiments are given below:

- For C. capitata 10X Genomics linked read and Nanopore sequencing, high-molecular-weight DNA was extracted from a pool of twenty individuals of each sex and strain.
For C. capitata Illumina sequencing, DNA was extracted from individual flies.
For C. capitata PacBio Sequel, a line was created with single pair crossing and subsequent sibling-mating for six generations, reared on tetracyclin-containing diet. HMW DNA was extracted from a pool of five males.
Phase GenomicsTM Hi-C libraries were made by Phase genomics from males (n=2) of the same family used for PacBio sequencing.

- Whole genome shotgun sequencing was performed on one single male and one female for B. dorsalis (white pupae),B. tryoni (Ourimbah strain, brown pupae), the Bactrocera introgression line (BIL, white pupae) and two pools of four BIL. High levels of nucleotide divergence exist between B. dorsalis and B. tryoni and therefore only few individuals were required for sequencing.

- RNAseq was carried out on 3rd instar and pre-pupa libraries in triplicates for C. capitata, B. dorsalis, and Z. cucurbitae. Sequencing six libraries per species, three at each stage, is sufficient to confirm and identify deletions. As the white pupa strains were founded by single individuals, the mutation causing the phenotype would be identical between all individuals allowing a smaller sample size to be used.

in situ hybridizations
- In situ hybridizations were done at least in duplicate and at least ten nuclei were analyzed per sample.

Injections
- Injected cohort size was determined for both B. tryoni and C. capitata based on previous work, referenced in text, to carry out NHEJ in both species:
Sample sizes of embryos for C. capitata and B. tryoni CRISPR injections were selected to ensure acceptable (~5%) survivorship based on previously carried out CRISPR experiments (C. capitata: Aumann et al., 2018 IBMB; B. tryoni: Choo et al., 2018 J. Appl. Entomol. 142, 52-58).
CRISPR/Cas9 injection into 588 C. capitata embryos produced 63 survivors (9.3%), all with brown puparium.

CRISPR/Cas9 injections into B. tryoni embryos were carried out over multiple times days. In total, 591 embryos were injected with two RNA guides targeting the first protein coding exon of the MFS gene. In total, 19 (3.2%) individuals survived and completed development, and presented with either wild type brown puparium (n = 12) or were mosaic with a somatic white-brown puparium (n = 7).

Data exclusions

No data was excluded

Replication

Replication was carried out by generating multiple mutant lines in both, B. tryoni and C. capitata. Multiple individuals containing different mutations were used to generate pure breeding white pupa lines:
Bactrocera tryoni CRIPSR/Cas9: Five crosses involving G0 survivors were fertile and produced G1 progeny. After crossing G1 siblings, three of these lines produced individuals with white pupae. Four different mutations were identified among the white pupa individuals through Sanger sequencing.
C. capitata: six G0 survivors were crossed individually, the remaining G0 flies were crossed in seven groups of seven to ten flies. Five out of 13 crosses produced white pupae phenotype G1 offspring (reciprocal crosses). Eight different mutations were identified through Sanger sequencing.
RNAseq libraries used to identify and confirm mutations were sequenced from replicate libraries containing different individuals.

In situ hybridizations were done at least in duplicate and at least ten nuclei were analyzed per sample.

PCRs on the D53 inversion breakpoints (C. capitata) were done at least twice, PCRs for non-lethal genotyping (knock-out experiment in C. capitata) were done once per individual, wp-CRISPR alleles were verified via sequencing.

All attempts at replication were successful.

Randomization

Individuals for sequencing, larvae for in situ, and embryos for injections were sampled from laboratory stocks in a randomized way.

Comparisons between species in both WGS, whole genome comparative and RNAseq comparisons carried out in this paper were a posteriori due to the nature of the experiments and comparisons being made. No other randomization was necessary due to the experiments requiring the sequencing and in situ analysis of targeted species and strains.

Blinding

Blinding was carried out when WGS samples were mapped to the reference, sample names were changed before genotyping. Blinding was not necessary for the experiments carried out to identify the introgressed region as populations needed to be known ahead of time to carry out tests.

Reporting for specific materials, systems and methods

We require information from authors about some types of materials, experimental systems and methods used in many studies. Here, indicate whether each material, system or method listed is relevant to your study. If you are not sure if a list item applies to your research, read the appropriate section before selecting a response.

Materials & experimental systems

n/a	Involved in the study	
x		Antibodies
x		Eukaryotic cell lines
x		Palaeontology and archaeology
	x	Animals and other organisms
x		Human research participants
x		Clinical data
x		Dual use research of concern

Methods

n/a	Involved in the study	
x		ChIP-seq
x		Flow cytometry
x		MRI-based neuroimaging

Animals and other organisms

Policy information about studies involving animals; ARRIVE guidelines recommended for reporting animal research

Laboratory animals	Laboratory reared flies of the species Bactrocera dorsalis, Bactrocera tryoni, Ceratitis capitata and Zeugodacus cucurbitae were used in this study. Strains and their usage are specified in the Supplementary Table 1.<br><br>Age of strains in generations:<br><br>Ceratitis capitata Egypt II: 420<br>C. capitata 1402_22m1B: ~110<br>C. capitata Benakeion: 78<br>C. capitata wp/tsl (in EgII): 340<br>C. capitata D53 (in wp/tsl EgII): 220<br>C. capitata Vienna 7 D53-: 68<br>C. capitata Vienna 8 D53-: 94<br><br>Bactrocera dorsalis Saramburi: 85<br>B. dorsalis white pupae: 33<br>B. tryoni Ourimbah: 47<br>BIL: ~ 10<br><br>Zeugodacus cucurbitae white pupae GSS: 102
Wild animals	This study did not involve wild animals.
Field-collected samples	This study did not involve field-collected samples.
Ethics oversight	All studies were conducted using insects only. There is no ethical assessments needed for such studies in Germany, Austria and Australia.

Note that full information on the approval of the study protocol must also be provided in the manuscript.
